# Polymicrobial Bacteremia Involving *Comamonas testosteroni*

**DOI:** 10.1155/2014/578127

**Published:** 2014-12-28

**Authors:** Jose Orsini, Eric Tam, Naomi Hauser, Salil Rajayer

**Affiliations:** ^1^Department of Medicine, New York University School of Medicine, Woodhull Medical and Mental Health Center, Brooklyn, NY 11206, USA; ^2^Division of Critical Care Medicine, Department of Medicine, New York University School of Medicine, Woodhull Medical and Mental Health Center, Brooklyn, NY 11206, USA

## Abstract

*Comamonas* spp. are uncommon isolates in microbiology laboratories and have been rarely observed as an infectious agent in clinical practice. They have widespread environmental distribution and have been isolated from water, soil, and plants as well as from some hospital devices such as intravenous catheters and water contained in humidifier reservoirs used in respiratory treatment. The genus *Comamonas* originally contained the following species: *acidovorans, testosteroni, kerstersii, terrigena, denitrificans*, and *nitrativorans*. It now contains 17 species, while *acidovorans* spp. have been reclassified as *Delftia acidovorans*. In spite of its uncommon human pathogenesis, there are few reports on the aggressive manner of it as an opportunistic pathogen, mostly related to *testosteroni* spp. We present a case of polymicrobial bacteremia involving *Comamonas testosteroni*. The aim of this case report is to alert clinicians to the potential diagnosis of bloodstream infections caused by uncommon pathogens.

## 1. Introduction

*Comamonas testosteroni*, formerly known as* Pseudomonas testosteroni*, is an aerobic, motile, nonspore forming, ubiquitous Gram-negative organism. Although it has low virulence potency, there are few reports on its involvement in human infections. It became clinically important after 1987, when reports began accumulating on human infections such as cellulitis [1], peritonitis [2], endocarditis [3], meningitis [4], endophthalmitis [5], tenosynovitis [6], and pneumonia [7]. However, cases of bloodstream infections caused by* Comamonas testosteroni* have been infrequently reported [2, 8–12]. The name of* testosteroni* was given due to its characteristic of utilizing carbon from the metabolism of testosterone [13], although this property has also been demonstrated by some* Pseudomonas *spp. and fungi [14]. Some strains of* Comamonas testosteroni* have acquired the plasmid-mediated* bla*NDM1, gene that confers broad spectrum antimicrobial resistance thus potentially hampering treatment options in the event where this bacterium causes human infections [15]. In this report, we present an uncommon case of polymicrobial bacteremia involving* Comamonas testosteroni* with a detailed review of the literature.

## 2. Case Report

An 80-year-old African American female, morbidly obese, was brought to emergency department complaining of generalized body aches (predominantly in shoulders) and nondocumented fevers at home for about 1 week. Her past medical history was significant for systemic arterial hypertension, diabetes mellitus, hiatal hernia, osteoarthritis, and cholelithiasis (s/p cholecystectomy). On arrival to the hospital, her vital signs were as follows: blood pressure of 150/77 mmHg, a regular heart rate of a 106 beats/minute, a respiratory rate of 21 breaths/minute, a temperature of a 100.8°F, and an oxygen saturation of 96% breathing ambient air.

Remarkable laboratory findings on admission were a white blood cell (WBC) count of 14, 500/mm^3^ (4.8–10.8), a sodium level of 129 mmol/L (135–147), a potassium level of 5.5 mmol/L (3.5–5.3), and a creatinine level of 2.0 mg/dL (0.8–2.0). C-reactive protein (CRP) and erythrosedimentation rate (ESR) levels were both elevated at 152.7 mg/L (1.0–4.0) and 130 mm/h (0–20), respectively. Her urine analysis showed positive leukoesterase and a WBC count of 10–15/hpf (0–5). Coagulation and liver profiles were within normal limits. Bilateral shoulder X-ray demonstrated degenerative changes, with a right rotator cuff tendinitis but no fractures. Chest radiography revealed no infiltrates.

She was admitted to the medicine ward with the presumptive diagnosis of urinary tract infection (UTI), and empiric antimicrobial therapy was initiated with ceftriaxone 2 grams intravenously daily. Blood cultures drawn on admission grew methicillin-sensitive* Staphylococcus aureus*, and antimicrobial therapy was stream lined to nafcillin 2 grams intravenously every 4 hours. Transthoracic echocardiogram (TTE) showed no vegetations. On day 3 of admission, her hospital course was complicated by worsening leukocytosis and a transient episode of hypotension which responded to intravenous fluids therapy. At that time, a new set of blood cultures was obtained from which* Comamonas testosteroni *was isolated (sensitive to ceftazidime, carbapenems, piperacillin/tazobactam, and trimethoprim/sulfamethoxazole). Antimicrobial therapy was then escalated to cefazolin 1 gram intravenously every 8 hours plus doripenem 250 mg intravenously every 8 hours. Abdomen, pelvis, and chest computed tomography (CT) were obtained, demonstrating the presence of diverticulosis but no abscess formation or mass. Repeated blood cultures were negative, and the patient was discharged home to complete a total of 4-week course of intravenous antimicrobial therapy.

## 3. Discussion

This is one of fewer than 15 cases of* Comamonas testosteroni* bacteremia reported in the literature and the first reported case of* Comamonas testosteroni* and* Staphylococcus aureus* polymicrobial bloodstream infection, according to a MEDLINE search using the words “*Comamonas*” and “bacteremia” and “bloodstream infection.” Approximately 35 cases of infections involving* Comamonas testosteroni* have been reported in the medical literature. Majority of infections are community-acquired rather than nosocomial [3, 5], and most of the patients reported had some degree of immunosuppression such as malignancy, chronic liver disease, and end-stage renal disease requiring hemodialysis [1, 11, 12]. Bacterial translocation from the gastrointestinal tract seems to play an important role in the pathogenesis of infections caused by* Comamonas *spp. [9, 16].

Based on results from previous reports, majority of the 14 reported patients with bacteremia caused by* Comamonas testosteroni* were males, their median age was 47 years (range, newborn–89), only one patient died, and predisposing factors for infection were not identified in two of them. Most of the patients responded well to appropriate antimicrobial therapy [12].* Comamonas* spp. isolates are usually susceptible to aminoglycosides, fluoroquinolones, carbapenems, piperacillin-tazobactam, cephalosporins, and trimethoprim-sulfamethoxazole [1, 4].

The patient discussed in this report did not have any obvious source of infection. She did not have central venous catheters, and the abdominal imaging did not evidence any acute inflammatory process or mass. Her immunosuppressive state was limited to diabetes mellitus, which might have played a role as a possible predisposing factor. Therefore, the authors hypothesized that the most likely source might have been the right shoulder rotator cuff tendinitis. Although this hypothesis cannot be proven, it is supported by the fact that the patient reported shoulder pain, the CRP and ESR levels were markedly elevated, and the right shoulder imaging showed rotator cuff tendinitis. In 2000, Isotalo et al. reported a case of polymicrobial tenosynovitis, involving an organism most likely to be related to* Comamonas *spp. based on biochemical factors [6]. If proven, our patient may have been only the second case of tenosynovitis caused by* Comamonas *spp. reported in the literature. We also postulate that the second likely mode of bacteremia in this case could have been dissemination of* Comamonas testosteroni* from the bowel following gastrointestinal translocation, given the finding of extensive diverticulosis demonstrated in the abdominal imaging.

Polymicrobial bacteremia involving* Comamonas testosteroni* have been reported in association with* Streptococcus parasanguis* and* Ralstonia pickettii *[17]. Two other cases of polymicrobial bacteremia involving* Comamonas* spp. (*Delftia acidovorans* (formerly known as* Comamonas acidovorans*) and* Comamonas kerstersii*) were previously reported in association with* Streptococcus agalactiae* and* Bacteroides fragilis*, respectively [17]. To our knowledge, this is the first case of bloodstream infection involving* Comamonas testosteroni* and* Staphylococcus aureus*.

In this report, we have highlighted an unusual cause of bloodstream infection.* Comamonas* spp. should be kept in mind as a rare cause of bacteremia.
